# Photocatalytic NO_x_ Removal Performance of TiO_2_-Coated Permeable Concrete: Laboratory Optimization and Field Demonstration

**DOI:** 10.3390/ma19010148

**Published:** 2025-12-31

**Authors:** Han-Na Kim, Hyeok-Jung Kim

**Affiliations:** 1 Carbon-Neutral Road Construction Technology Research Institute, 62, Cheongsu 14-ro, Dongnam-gu, Cheonan-si 31198, Chungcheongnam-do, Republic of Korea; hnk07@hanmail.net; 2 Division of Architecture Engineering and Civil Engineering, Hoseo University, 20, Hoseo-ro 79beon-gil, Baebang-eup, Asan-si 31499, Chungcheongnam-do, Republic of Korea

**Keywords:** photocatalytic NO_x_ removal, TiO_2_ coating: permeable concrete, accelerated weathering resistance, mock-up test, field demonstration

## Abstract

Nitrogen oxides (NO_x_) emitted mainly from vehicle exhaust significantly contribute to urban air pollution, leading to photochemical smog and secondary particulate matter. Photocatalytic technology has emerged as a promising solution for continuous NO_x_ decomposition under ultraviolet (UV) irradiation. This study developed an eco-friendly permeable concrete incorporating activated loess and zeolite to improve roadside air quality. The high porosity and adsorption capability of the concrete provided a suitable substrate for a TiO_2_-based photocatalytic coating. A single-component coating system was optimized by introducing colloidal silica to enhance TiO_2_ particle dispersibility and adding a binder to secure durable adhesion on the concrete surface. The produced permeable concrete met sidewalk quality standards specified in SPS-F-KSPIC-001-2006. Photocatalytic NO_x_ removal performance evaluated by ISO 22197-1 showed a maximum removal efficiency of 77.5%. Even after 300 h of accelerated weathering, the activity loss remained within 13.8%, retaining approximately 80% of the initial performance. Additionally, outdoor mock-up testing under natural light confirmed NO_x_ concentration removal and formation of nitrate by-products, demonstrating practical applicability in real environments. Overall, the integration of permeable concrete and a durable, single-component TiO_2_ photocatalytic coating provides a promising approach to simultaneously enhance pavement sustainability and reduce urban NO_x_ pollution.

## 1. Introduction

Air pollution in modern urban environments has been exacerbated by rapid industrialization and increased traffic volumes, with nitrogen oxides (NO_x_) from vehicle exhaust being recognized as one of the major pollutants. NO_x_ acts as a precursor to photochemical smog and contributes to the formation of secondary fine particulate matter (PM_2_._5_) [1]. In the atmosphere, NO_x_ reacts with ozone (O_3_) to produce photochemical oxidants [2,3], which can cause respiratory and cardiovascular diseases, thereby posing serious risks to human health and ecosystems [4,5]. In response, various policies and technologies have been developed to reduce NO_x_ emissions; however, most efforts have focused on emission control at the source or end-of-pipe treatments, which have limitations in addressing residual pollutants within urban environments [6,7].

As a novel approach to mitigating urban air pollution, technologies that impart photocatalytic functions to urban infrastructure surfaces—such as sidewalks, bicycle paths, and building façades—have attracted considerable attention [8,9]. Among various photocatalysts, titanium dioxide (TiO_2_) is widely recognized for its cost-effectiveness and environmental compatibility. Under ultraviolet (UV) irradiation, TiO_2_ generates electron–hole pairs (e^−^/h^+^) on its photoactive surface, which subsequently produce reactive oxygen species (ROS) such as superoxide radicals (·O_2_^−^) and hydroxyl radicals (·OH). These reactive species oxidize NO_x_ into non-volatile nitrate (NO_3_^−^), which can either remain fixed on the surface or be removed by rainfall, thereby contributing to the removal of NO_x_ concentrations in the atmosphere [10,11].

Previous applications of TiO_2_ photocatalysts have primarily focused on evaluating the potential for NO_x_ removal by coating or incorporating them into impermeable substrates such as concrete and asphalt. However, these substrates exhibit low porosity, which restricts the available surface area for photocatalyst attachment [12,13]. This limitation hinders the sufficient exposure of active sites, thereby reducing reaction efficiency. In addition, external environmental factors such as vehicular traffic, pedestrian activity, and weathering frequently lead to surface abrasion, resulting in a decline in the durability of the photocatalytic layer [14]. In particular, detachment or cracking of the photocatalytic coating causes long-term deterioration of NO_x_ removal performance, making it challenging to maintain effectiveness under outdoor conditions [15,16]. These issues strongly underscore the need for developing novel substrate materials with higher porosity and enhanced durability, along with advanced coating stabilization techniques [17].

To overcome these limitations, the use of natural microporous materials such as activated loess and zeolite has gained increasing attention. Activated loess, with its high specific surface area and excellent pozzolanic reactivity, has been investigated as a potential cement substitute, while zeolite, owing to its well-defined microporous structure, demonstrates strong adsorption capacity for hazardous gases and pollutants [18,19]. When incorporated into permeable concrete, these materials are expected to simultaneously enhance porosity and adsorption performance. Nevertheless, research on applying photocatalysts to such porous substrates remains at an early stage. In particular, there is a lack of empirical studies investigating the synergistic effects between the high porosity of permeable concrete and photocatalytic activity. Moreover, systematic evaluations addressing critical issues such as improving adhesion between photocatalysts and substrates, stabilizing coatings, and ensuring long-term durability under outdoor environmental conditions are still insufficient [10].

Enhancing the functionality of construction materials has emerged as an important research field to address climate change and mitigate environmental challenges in urban areas. In particular, permeable concrete facilitates rainwater infiltration, thereby improving urban water circulation systems and reducing the risk of flooding. Furthermore, its evaporative cooling effect has been reported to alleviate the urban heat island phenomenon [20,21,22,23]. In addition, permeable concrete possesses a relatively higher porosity compared to conventional impermeable concrete, offering the potential to expand the effective reactive surface area of functional coatings applied to its surface [24]. These characteristics can expand the active surface area of the photocatalytic coating, thereby increasing the frequency of interactions with airborne pollutants and enhancing photocatalytic reactivity [25,26]. Therefore, applying TiO_2_ photocatalysts to eco-friendly permeable concrete incorporating activated loess and zeolite enables the development of a novel urban infrastructure material that simultaneously ensures eco-friendliness, high porosity, adsorption capacity, and photocatalytic activity. To secure the long-term performance of such photocatalytic coatings, it is essential to achieve uniform dispersion of TiO_2_ particles, improve coating adhesion, and ensure weather resistance under outdoor exposure conditions.

In this study, permeable concrete incorporating activated loess and zeolite was developed as a sustainable substrate, and a single-component TiO_2_ photocatalytic coating was optimized for NO_x_ removal performance. To improve the dispersibility of TiO_2_ particles and enhance adhesion to the concrete surface, colloidal silica was introduced, while an acrylic binder was employed to further improve coating durability under outdoor conditions. The effects of these additives on photocatalytic performance and weather resistance were systematically evaluated through both laboratory-scale experiments and simulated road environment tests. The findings of this study aim to establish design guidelines for durable and high-performance photocatalytic pavements, thereby contributing to the development of next-generation urban air quality improvement technologies.

## 2. Materials and Methods

Automobile exhaust in urban areas is a major source of nitrogen oxides (NO_x_), which continue to raise concerns due to their contribution to photochemical smog and fine particulate matter. As a countermeasure, increasing attention has been directed toward technologies that impart photocatalytic functions to infrastructure surfaces, such as sidewalks and building façades, to induce environmental purification effects. In this study, eco-friendly permeable concrete incorporating activated loess and zeolite was employed as a substrate, onto which a photocatalytic coating was applied. The composition of the photocatalytic coating was optimized, and the NO_x_ removal efficiency was experimentally verified under both laboratory and outdoor conditions to evaluate its practical applicability. In particular, colloidal silica and a binder were introduced to enhance the dispersion stability of TiO_2_ particles within the single-component coating and to improve adhesion to the substrate, while the effects of varying their contents on NO_x_ removal performance were systematically analyzed.

### 2.1. Materials

#### 2.1.1. Permeable Concrete

**Figure 1:**
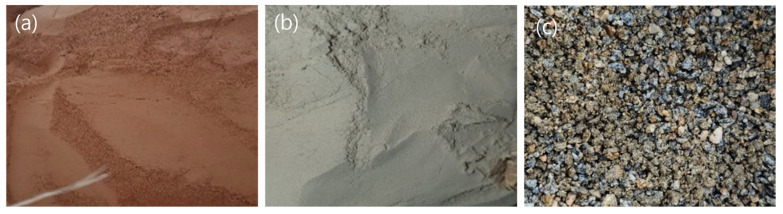
Images of (**a**) active loess, (**b**) zeolite and (**c**) gravel.

The cement used in this study was Type I ordinary Portland cement (OPC) manufactured in Korea, while activated loess and zeolite were employed as eco-friendly supplementary materials to partially replace OPC (Figure 1). OPC was selected as the primary binder in this study due to its widespread availability, standardized performance, and suitability for pavement concrete applications. OPC is the most used cement in sidewalk and pavement construction, as it provides stable early-age strength development and predictable hydration behavior under normal curing conditions. These characteristics are particularly important for permeable concrete, where sufficient early strength is required to secure aggregate interlocking while maintaining a high interconnected porosity. The physical properties of OPC, activated loess, and zeolite used in the permeable concrete mixture are presented in Table 1, and their chemical compositions are summarized in Table 2. OPC is characterized by a high CaO content, as summarized in Table 2, which governs its hydration reactions and contributes to the formation of calcium silicate hydrate (C–S–H) and calcium hydroxide (Ca(OH)_2_). This CaO-dominated hydration mechanism ensures adequate mechanical performance and structural integrity of the permeable concrete matrix, even when part of the cement is replaced by supplementary materials. Activated loess and zeolite a contain high proportions of SiO_2_ and Al_2_O_3_ and exhibit relatively low densities and high specific surface areas compared to OPC. These characteristics promote pozzolanic reactions between the amorphous silica/alumina phases of the supplementary materials and the calcium hydroxide generated during OPC hydration. Through this pozzolanic reaction, secondary C–S–H and calcium aluminate hydrate (C–A–H) phases are formed, contributing to long-term strength development and microstructural densification of the binder matrix. At the same time, the inherently porous structure of activated loess and zeolite enhances their adsorption capacity for gaseous pollutants.

The coarse aggregate used in this study complied with the KS F 2527 standard [27] and consisted of single-graded aggregate with particle sizes between 10 and 13 mm (Figure 1). This aggregate was selected to ensure sufficient porosity and strength development in permeable concrete. Prior to use, the aggregate was washed to remove surface impurities.

**Table 1:** Physical properties of cement, active loess and zeolite [28].

Type	Density (g/cm^3^)	Blaine (cm^2^/g)
Cement	3.15	3000
Active loess	1.92	3000
Zeolite	2.50	3300

**Table 2:** Chemical composition of cement, active loess and zeolite [28].

Type	SiO_2_	Al_2_O_3_	Fe_2_O_3_	CaO	K_2_O	MgO	Etc.
Cement	21.9	4.8	3.4	62.6	-	2.6	4.7
Active loess	43.0	35.9	10.8	7.2	0.8	1.6	1.7
Zeolite	68.9	16.4	5.3	2.6	3.7	1.0	2.1

#### 2.1.2. TiO_2_ Photocatalyst

For imparting NO_x_ removal performance to the permeable concrete, a TiO_2_ photocatalyst with an anatase crystal structure (AERODISP^®^ W740X, Evonik industries, Essen, Germany) was used. AERODISP^®^ W740X is a hydrophilic, aqueous dispersion of fumed titanium dioxide with a solid content of 40%. Its physicochemical properties are summarized in Table 3.

**Table 3:** Physico-chemical properties of TiO_2_ photocatalyst [29].

Properties	Unit	Value
TiO_2_ content	%	39–41
Viscosity	mPas	≤30
pH value		5.0–7.0
Density at 20 °C	g/cm^3^	1.41
Particle size	nm	70

To ensure the stable adhesion of TiO_2_ particles onto the concrete surface, two types of binder materials were employed: R-709 (a butyl acrylate–methyl acrylate copolymer, Mokbon Industry, Yangsan, Republic of Korea) and SS-SOL 30 (colloidal silica, S-CHEMTECH, Ansan, Republic of Korea). These additives were incorporated to improve coating adhesion and provide long-term durability under outdoor exposure conditions.

### 2.2. Experimental Variable

The mix proportions of the permeable concrete used in this study are summarized in Table 4. The water-to-binder ratio (W/B) was fixed at 36.4% for all mixtures. According to previous studies, when the replacement level of porous pozzolanic materials exceeds 15% of cement, the compressive strength and durability of permeable concrete tend to decrease significantly, whereas the combination of activated loess and zeolite at a 1:1 ratio yielded the most favorable physical performance [10]. Based on these findings, the total pozzolanic replacement ratio was fixed at 15% of cement, with activated loess and zeolite incorporated at a 1:1 ratio in the experimental design. In Table 4, the specimen code “CTRL” denotes the control mixture in which 100% cement was used as the binder without any supplementary materials, whereas “A1Z1” refers to the blended binder mixture in which 7.5% activated loess and 7.5% zeolite were substituted for cement.

According to previous studies, the photocatalytic coating using AERODISP^®^ W740X exhibited the highest NO_x_ removal performance when the TiO_2_ content was 7.5% [10]. Accordingly, in this study, the TiO_2_ content was fixed at 7.5%, and experiments were conducted by varying the amounts of SS-SOL and R-709 to determine the optimal formulation of the coating. The mix proportions of the photocatalytic coatings are presented in Table 5.

### 2.3. Characterization

#### 2.3.1. Mechanical and Durability Tests

The mechanical and durability performance of the permeable concrete for pavement applications was evaluated in accordance with the SPS-F-KSPIC-001-2006 [30] standard. This standard specifies different performance requirements depending on the application, including sidewalks, bicycle paths, and parking lots. It defines the minimum performance criteria for compressive strength, flexural strength, residual compressive strength after 100 freeze–thaw cycles, skid resistance, and permeability coefficient. The required performance values for each application are summarized in Table 6. Each test was conducted in accordance with the following standards. Compressive strength was measured by uniaxial compression testing following KS F 2405 [31]. Flexural strength, skid resistance, and permeability coefficient were evaluated in accordance with KS F 2408 [32], KS F 2375 [33], and KS F 4001 [34], respectively. Freeze–thaw resistance was assessed according to KS F 2456 [35], in which 100 freeze–thaw cycles were applied, and the residual compressive strength was subsequently measured in accordance with KS F 2405.

#### 2.3.2. NO_x_ Removal Performance Evaluation

**Figure 2:**
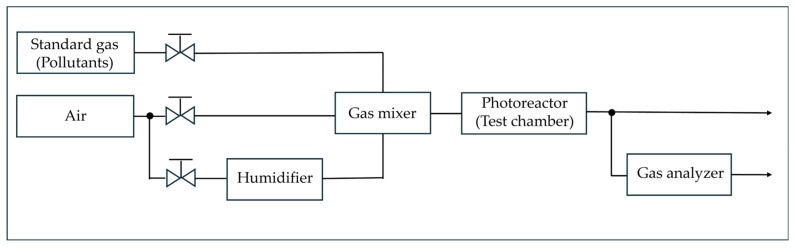
Schematic diagram of the NO_x_ removal performance evaluation system.

**Figure 3:**
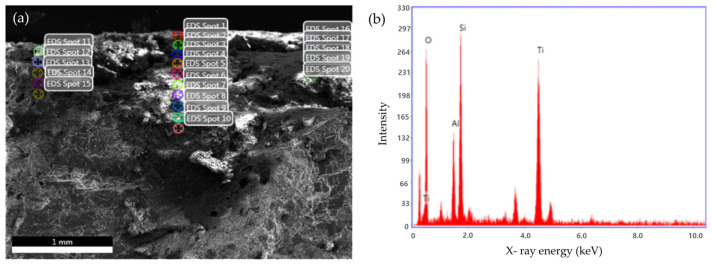
SEM images (**a**) and EDS spectra (**b**) of a TiO_2_-cated concrete cross-section.

The NO_x_ removal performance of the photocatalyst was evaluated in accordance with the KS L ISO 22197-1:2016 standard test method [36]. As shown in Figure 2, the NO_x_ removal evaluation system was presented in two parts: (a) a schematic diagram and (b) a photograph of the apparatus. Permeable concrete specimens with dimensions of 50 mm × 10 mm × 25 mm were prepared for testing. To prevent photocatalytic reactions on the lateral surfaces during the experiment, the sides of the specimens were sealed with an inert material. Prior to testing, the specimens were immersed in deionized water for more than 2 h, followed by drying in an oven at 40 °C for at least 24 h as a pretreatment process. Subsequently, the prepared photocatalyst was uniformly applied to the specimen surfaces using an automatic spray device. The TiO_2_ coating was applied using an automated XY-programmed spray system (Delta controller), which ensured uniform and repeatable coating coverage over the exposed concrete surface. The coating area was set to 100 mm × 50 mm, with the spray path controlled between X = 20–120 mm and Y = 20–70 mm. The spray interval was fixed at 10 mm, with one complete XY scan per coating cycle. The traverse speed of the spray head was maintained at 5 mm/s, and the air flow rate supplied to the spray nozzle was set to 2.5 (instrument setting unit), ensuring stable atomization. The amount of TiO_2_ coating applied to each specimen was quantified by measuring the mass of the specimens before and after spraying, and the net coating mass was calculated by subtracting the initial specimen mass from the final mass after complete drying. The average TiO_2_ loading was determined to be approximately 32.25 ± 0.75 g/m^2^, assuming uniform deposition over the exposed surface area. To verify that the photocatalytic material was uniformly and reliably applied to the concrete surface, the cross-sectional morphology and elemental distribution of the coated specimens were examined prior to the NO_x_ removal experiments. As presented in Figure 3a, the SEM image clearly shows the formation of a continuous photocatalyst layer conformally covering the concrete surface without noticeable peeling, voids, or delamination. The corresponding EDS spectra confirm the presence of Ti-derived signals. Figure 3b presents the EDS spectra obtained from the cross-section of the TiO_2_-coated permeable concrete. A distinct Ti peak is clearly observed together with signals from the cementitious matrix, indicating the presence of TiO_2_ on the pavement surface. This confirms that the photocatalyst was successfully deposited onto the concrete substrate rather than being locally deficient or detached. Combined with the SEM observations, these results verify that a continuous and well-adhered TiO_2_; coating layer was formed, providing a reliable active surface for subsequent NO_x_ removal performance evaluation. For the test, 1 ppm NO gas was used, prepared by mixing standard NO gas with either dry or humidified air. The specimens were placed inside the test chamber, where the conditions were maintained at a temperature of 25 ± 1 °C, relative humidity of 50 ± 5%, and a gas flow rate of 3 L/min. The test was conducted for a total duration of 7 h. First, the test gas was introduced for 1 h to allow stabilization. Subsequently, ultraviolet irradiation was applied for 5 h using a UV-A lamp with an intensity of 1 mW/cm^2^ as the light source. Finally, after switching off the UV light, changes in the gas concentration were monitored for an additional 1 h. During each NOx reduction test, the outlet NO concentration was continuously recorded as an absolute value (ppm) using the NO_x_ analyzer. For comparative interpretation, the temporal NO concentration profiles were normalized by defining the stabilized NO concentration immediately before UV irradiation as 100%. The NO_x_ removal efficiency was calculated using the following equation (Equation (1)).

(1) $${NO}_{x}\;removal\;efficiency\left(\%\right)=\frac{\left({NO}_{x,initial}-{NO}_{x,equil}\right)}{{NO}_{x,equiil}}\times 100$$

here, ${NO}_{x,initial}$ represents the initial NO_x_ concentration at the beginning of the test, and ${NO}_{x,equil}$ denotes the NO_x_ concentration measured at the equilibrium state during UV irradiation.

#### 2.3.3. Accelerated Weathering Test

**Figure A1:**
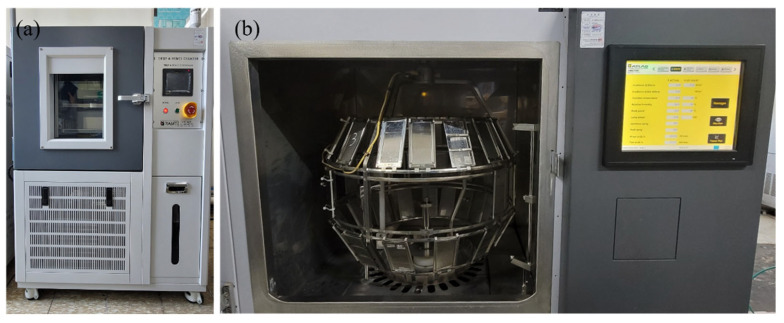
Photographs of the accelerated weathering test chamber: (**a**) exterior and (**b**) interior view.

To evaluate the long-term performance and durability of the photocatalytic coating, accelerated weathering tests were conducted in accordance with the KS M ISO 4892-2:2013 standard [37]. Permeable concrete specimens prepared for NO_x_ removal performance evaluation were used for this test. The specimens were placed inside the chamber of the accelerated weathering apparatus, and the test was carried out for a total of 300 h. The apparatus used for the accelerated weathering test is shown in Figure A1, and the detailed test conditions are summarized in Table 7. To quantitatively evaluate the durability changes in the photocatalytic coating, the following parameters were compared before and after the accelerated weathering test. First, the surface appearance of the photocatalyst was examined in accordance with KS M ISO 4892-2:2013, and the degree of discoloration was assessed using the Gray Scale specified in KS M ISO 105-A02:1993 [38]. In addition, the color difference (ΔE) was measured according to KS A 0063:2015 [39]. The change in NO_x_ removal efficiency of the photocatalytic coating was evaluated by comparing the NO_x_ removal performance of the specimens under identical conditions before and after the accelerated weathering test.

#### 2.3.4. Mock-Up-Based Evaluation of NO_x_ Removal Performance

**Figure A2:**
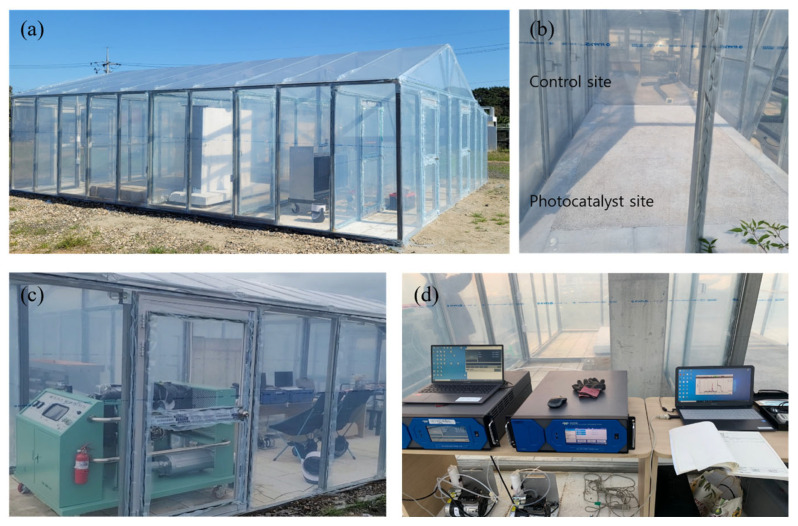
Configuration of the test-bed: (**a**) overall view of the test-bed, (**b**) eco-friendly permeable concrete section, (**c**) exhaust gas generation device, and (**d**) NO_x_ removal experiment control room.

In this study, the NO_x_ removal performance of photocatalyst-coated permeable concrete for sidewalk pavement was evaluated through a mock-up (test-bed) experiment designed to simulate real road conditions. The test was conducted at the Outdoor Demonstration Center of the Korea Conformity Laboratories (KCL). As shown in Figure A2a, a sealed test space was constructed using polyethylene (PE) film with a UV-A transmittance of approximately 60% to induce photocatalytic reactions under ultraviolet irradiation. The test-bed consisted of two sites: a control site with conventional permeable concrete pavement and a photocatalyst site of the same dimensions, in which eco-friendly permeable concrete coated with TiO_2_ photocatalyst was applied (Figure A2b). For the outdoor mock-up evaluation, the photocatalytic TiO_2_ coating was applied over a surface area of approximately 6 m^2^ (3000 mm × 2000 mm). The NO removal performance was analyzed within a test space having an internal volume of approximately 15 m^3^ (3000 mm × 2000 mm × 25,000 mm). To simulate air pollutants generated in actual road environments, an exhaust gas generator fabricated from an aged automobile engine was employed, and the exhaust gases produced were introduced into the test space (Figure A2c). After simultaneously injecting the polluted gas into the two sealed spaces, an internal circulation fan was operated to thoroughly mix the injected gas with the chamber air, thereby ensuring homogeneous NO concentration before monitoring. The NO_x_ removal performance was evaluated by partially modifying the test conditions based on the KS M ISO 4892-2:2013 standard. The initial NO gas concentration was set at 1000 ppb (1 ppm), and the time required for the concentration inside the test-bed to decrease to 100 ppb (0.1 ppm) was measured and comparatively analyzed between the control and photocatalyst sites. Real-time monitoring of NO concentrations was performed using a Serinus 40 NO_x_ analyzer (ADB, Sydney, Australia), which was installed in each test section as shown in Figure A2d. From a mass-balance perspective, the observed decay of NO concentration within the mock-up chamber reflects the combined effects of photocatalytic oxidation on the TiO_2_-coated surface and the defined airflow and mixing conditions inside the space, rather than representing an intrinsic reaction kinetic constant under ideal plug-flow or closed-photoreactor conditions. Therefore, the “time required to reduce the NO concentration to 100 ppb” is interpreted as a comparative performance indicator under identical chamber volume, mixing, and ventilation conditions, enabling a realistic comparison between different coating systems in a field-relevant environment.

## 3. Results and Discussion

### 3.1. Physical Properties of Permeable Concrete

Eco-friendly permeable concrete incorporating activated loess and zeolite was fabricated and evaluated according to the quality criteria for sidewalk permeable concrete specified in SPS-F-KSPIC-001-2006. The tests were conducted on both ordinary concrete (Control, CTRL) and the activated-loess–zeolite mixture concrete (A1Z1), and the major results are summarized in Table 8. At 28 days, the CTRL specimen exhibited a compressive strength of 22.5 MPa and a flexural strength of 3.9 MPa. In comparison, the A1Z1 specimen showed slightly reduced values—20.7 MPa in compressive strength (92.0% of CTRL) and 3.6 MPa in flexural strength (92.3% of CTRL). Nevertheless, both mixtures fully satisfied the minimum strength requirements for sidewalk applications (compressive strength ≥ 12 MPa and flexural strength ≥ 1.2 MPa). Freeze–thaw durability was also verified by measuring the residual compressive strength after 100 freeze–thaw cycles. The CTRL specimen retained 19.6 MPa (87.1% of its 28-day strength), while A1Z1 maintained 16.8 MPa (82.4%), meeting the required residual strength ratio of ≥80%. Skid resistance results showed values of 48.3 BPN for CTRL and 48.0 BPN for A1Z1, both exceeding the minimum standard of 30 BPN. Additionally, both mixtures demonstrated permeability coefficients greater than 1.0 × 10^−3^ cm/s, confirming that they possess sufficient permeability for sidewalk applications. Collectively, these results indicate that the permeable concrete containing activated loess and zeolite exhibits mechanical performance and durability comparable to conventional permeable concrete while maintaining the required quality standards.

### 3.2. Effect of Binder Addition on the NO Removal Performance of Single-Component TiO_2_ Photocatalysts

**Figure 4:**
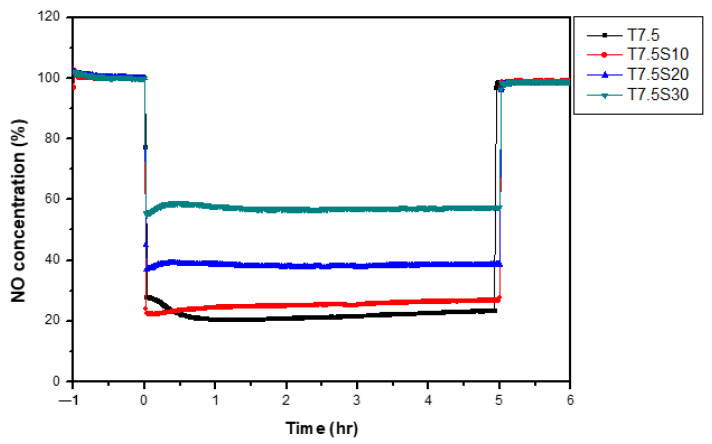
Changes in NO concentration according to colloidal silica content.

To enhance the adhesion of the photocatalytic coating to the concrete surface, single-component TiO_2_ coatings with different colloidal silica contents were prepared based on the optimal TiO_2_ content of 7.5%, identified in previous studies. The prepared photocatalysts were applied to A1Z1 permeable concrete substrates, and NO removal performance was evaluated according to ISO 22197-1:2016. Figure 4 compares NO concentration profiles for coatings with varying colloidal silica contents, while Table 9 summarizes the NO removal efficiencies. The baseline specimen (T7.5) exhibited the highest NO removal efficiency of 77.5%. However, the addition of colloidal silica resulted in a gradual decline in photocatalytic performance, with efficiencies decreasing to 74.4%, 61.8%, and 42.8% for S10, S20, and S30 mixtures, respectively. This decline is attributed to the partial shielding of active TiO_2_ surface sites and increased light scattering caused by colloidal silica, which limits UV penetration and reduces accessibility of NO_x_ molecules to photocatalytically active sites. The reduced photocatalytic performance of the TiO_2_–silica composite coating is attributed to the combined effects of partial light shielding and scattering caused by silica particles, as well as potential changes in surface accessibility. Although direct optical characterization such as UV transmittance or reflectance measurements and quantitative surface accessibility analyses was not conducted in the present study, this interpretation is supported by previously reported findings in the literature that silica incorporation can alter photon penetration and active-site exposure on photocatalyst surfaces. Future work will include detailed optical measurements and quantitative accessibility analyses to more rigorously validate this mechanism [40,41,42,43]. Although colloidal silica improves coating adhesion, excessive amounts negatively impact photocatalytic reactivity. Therefore, T7.5S10—which exhibited less than a 10% removal compared to T7.5—was selected as the optimal colloidal silica content.

**Figure 5:**
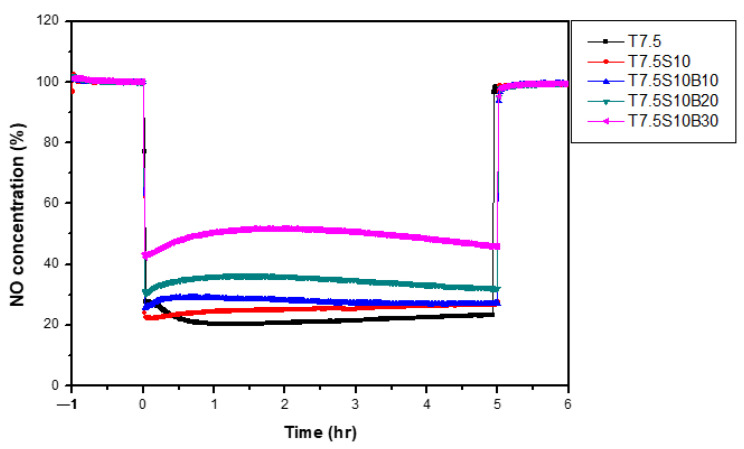
Changes in NO concentration according to binder content.

Subsequently, an acrylic binder was incorporated into the T7.5S10 formulation at varying contents to further enhance adhesion between TiO_2_ particles and the concrete surface. NO removal performance decreased progressively with increasing binder content (Figure 5, Table 10), as the binder partially encapsulates TiO_2_ particles and obstructs UV irradiation and gas–surface interactions. Nonetheless, the T7.5S10B20 specimen maintained a removal efficiency loss within 10% and was thus selected as the optimal binder condition for outdoor performance evaluation.

### 3.3. Accelerated Weathering Resistance

**Figure 6:**
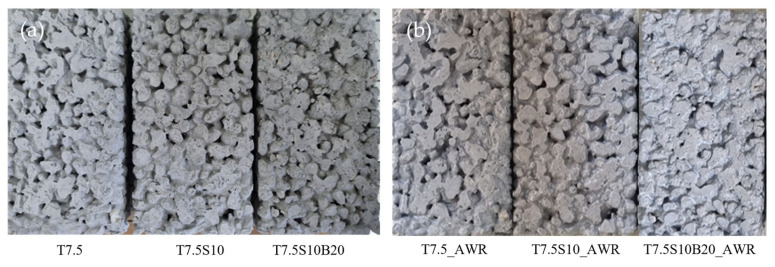
Changes in surface appearance and color characteristics of TiO_2_-coated permeable concrete specimens before (**a**) and after (**b**) accelerated weathering.

Accelerated weathering tests were conducted in accordance with KS M ISO 4892-2:2013 to assess the long-term durability of the single-component photocatalytic coatings under simulated outdoor conditions. This method exposes specimens to control UV radiation, heat, humidity, and water spray cycles, enabling prediction of long-term degradation within a shortened period. Coated permeable concrete samples were subjected to 300 h of accelerated weathering. Figure 6a,b show the surface appearance of the TiO_2_-coated permeable concrete specimens before and after the accelerated weathering test, respectively. As shown in Figure 6, no visible defects—such as cracking, peeling, or delamination—were observed on any specimens. The gray scale rating for discoloration remained at grade 4 across all samples, indicating minimal visual change. Color difference (ΔE) values ranged from 1.5 to 2.0, corresponding to changes imperceptible to the human eye. These results confirm that the single-component TiO_2_ coating maintains adequate physical and chemical stability under prolonged UV exposure and varied environmental conditions. The results of the accelerated weathering evaluation are summarized in Table 11.

**Figure 7:**
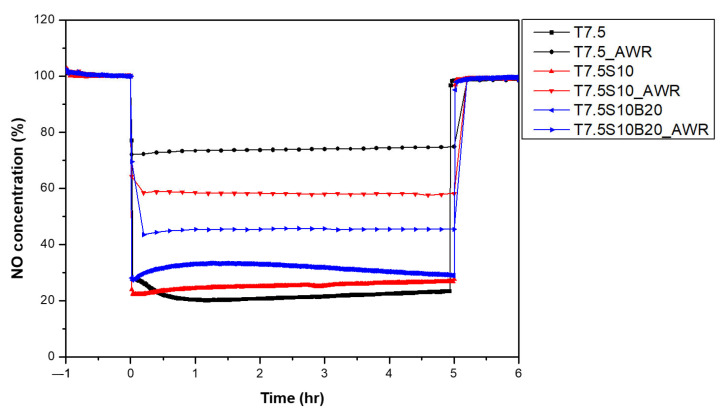
Comparison of photocatalytic NO removal performance before and after accelerated weathering.

Figure 7 and Table 12 present the changes in NO removal efficiency before and after accelerated weathering. The T7.5 specimen, which contained no adhesion-enhancing components, exhibited a significant decrease of 51.5% in photocatalytic efficiency after weathering—primarily due to particle detachment from the substrate. In contrast, T7.5S10 showed a smaller removal of 32.5%, attributed to stronger chemical bonding between TiO_2_ particles and the substrate induced by colloidal silica. The T7.5S10B20 specimen demonstrated the highest durability, retaining approximately 80% of its initial performance with only a 13.8% decrease after weathering. This result confirms that the acrylic binder plays a critical role in maintaining particle adhesion and minimizing performance deterioration under outdoor exposure.

### 3.4. Mock-Up Evaluation of TiO_2_-Coated Pavement

**Figure 8:**
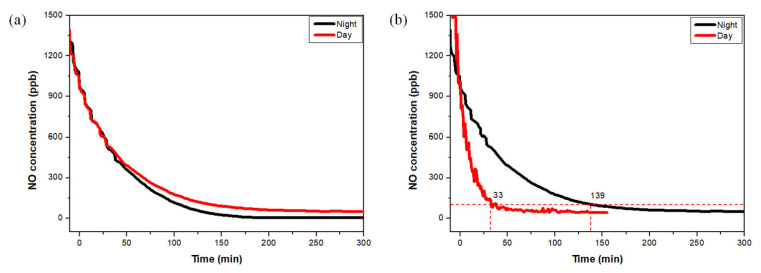
Changes in NO concentration depending on the presence or absence of photoreaction in general pavement (**a**) and photocatalyst coated pavement (**b**).

A full-scale mock-up experiment was conducted to validate the NO_x_ removal performance of the photocatalyst-coated permeable pavement under conditions simulating a real roadside environment. A modified diesel engine served as the emission source, supplying exhaust gases into a sealed test-bed. Variations in NO concentration were monitored under both daytime and nighttime conditions. Figure 8a shows that the uncoated pavement exhibited gradual removals in NO concentrations during both day and night. This was attributed to natural dilution caused by airflow within the test-bed and the measurement system’s exhaust mechanism, despite the absence of photocatalytic activity. In contrast, Figure 8b demonstrates that the photocatalyst-coated pavement showed a rapid decrease in NO concentration under daylight conditions due to activation of the TiO_2_ photocatalyst by natural UV irradiation. At night, the absence of UV light suppressed photocatalytic reactions, resulting in slower NO removal rates. These findings clearly demonstrate the UV-dependent nature of the photocatalytic process and confirm the enhanced NO_x_ removal capability of the coated pavement under realistic outdoor conditions.

### 3.5. Analysis of Nitrate Accumulation

**Figure 9:**
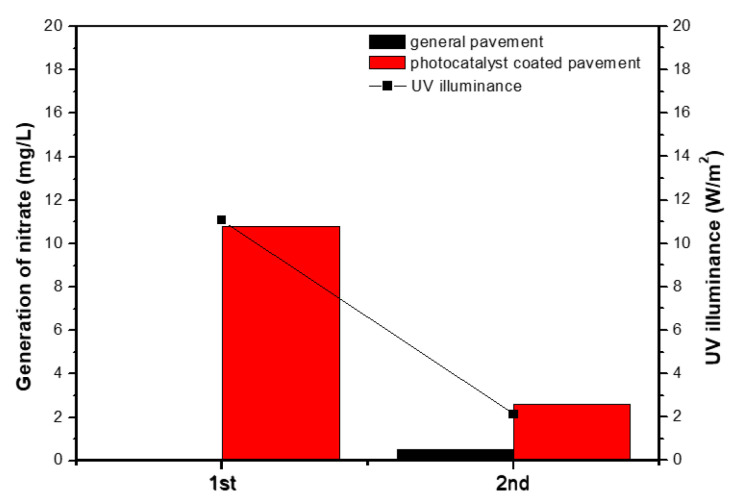
Comparison results of nitrate concentration according to illuminance in general pavement and photocatalyst coated pavement.

To indirectly confirm the photocatalytic oxidation of NO_x_, nitrate (NO_3_^−^) accumulation on pavement surfaces before and after testing was examined. Samples were collected by dissolving surface residues in ultrapure water, and nitrate concentrations were measured (Figure 9). Table 13 summarizes nitrate concentrations and corresponding UV illuminance levels recorded during the two mock-up experiments. On the uncoated pavement, nitrate formation was negligible (−0.1 to 0.5 mg/L), indicating the absence of photocatalytic oxidation. In contrast, the photocatalyst-coated pavement showed substantial nitrate accumulation ranging from 2.6 to 10.8 mg/L, providing indirect evidence of NO_x_ -to-nitrate conversion via TiO_2_ photocatalysis. A positive correlation was also observed between UV illuminance and nitrate generation, confirming that the photocatalytic oxidation mechanism is strongly dependent on UV intensity.

## 4. Conclusions

In this study, eco-friendly permeable concrete incorporating activated loess and zeolite was developed, and a single-component TiO_2_ photocatalyst coating was optimized and evaluated under both laboratory and simulated field conditions. The permeable concrete satisfied all quality requirements for sidewalk applications—including compressive strength, flexural strength, permeability, skid resistance, and freeze–thaw durability—demonstrating its suitability as a sustainable pavement substrate. After 300 h of accelerated weathering, the optimized coating retained approximately 80% of its initial NO_x_ removal efficiency, confirming its durability under prolonged UV exposure. In full-scale mock-up tests, the photocatalyst-coated pavement exhibited faster NO removal than uncoated pavement under natural sunlight, and nitrate accumulation analyses further verified the photocatalytic oxidation of NO_x_. These results demonstrate that integrating an activated-loess–zeolite permeable concrete substrate with an optimized single-component TiO_2_ photocatalyst coating offers strong potential for developing practical pavement materials aimed at improving urban air quality.

## Figures and Tables

**Table 4:** Mixing ratio of permeable concrete [10].

Specimen	W/B (%)	Water (kg)	Binder (kg)			Gravel (kg)
			Cement	Active Loess	Zeolite	
CTRL	36.4	120	330	-	-	1600
A1Z1			280	24.75	24.75	

**Table 5:** Mixing ratio of photocatalytic coating agent.

Sample	W740X (g)	SS-SOL (g)	R-709 (g)	Water (g)	Total (g)
T0	-	-	-	-	
T7.5	18.75	-	-	81.25	100
T7.5S10	18.75	1.88	-	79.37	100
T7.5S20	18.75	3.75	-	77.50	100
T7.5S30	18.75	5.62	-	75.63	100
T7.5S10B10	18.75	1.88	1.88	77.49	100
T7.5S10B20	18.75	1.88	3.75	75.62	100
T7.5S10B30	18.75	1.88	5.62	73.75	100

**Table 6:** Quality and requirement of permeable concrete for pavement.

	SideWalk	Bicycle Load	Parking Lot
Compressive Strength (MPa)	≥12	≥15	≥18
Flexural Strength (MPa)	≥1.2	≥1.5	≥1.8
Compressive Strength after 100 cycle freeze–thaw (%)	≥80% of strength at 28 days		
Skid Resistance (BPN)	≥30	≥40	≥40
Permeability Coefficient (cm/sec)	≥1.0 × 10^−3^		

**Table 7:** Accelerated weathering test conditions.

Item	Condition
Inner/outer filter	S.Borosilicare
Black panel temperature (test specimen surface temperature)	63 ± 3 °C
Relative humidity	50 °C ± 10%
Cycle	102 min of irradiation followed by 18 min of irradiation and water spray
Irradiance of the test specimen surface	0.51 W/m^2^ at 340 nm

**Table 8:** Summary of mechanical properties and durability of CTRL and A1Z1.

Item	Values		
	CTRL	A1Z1	Standard (Sidewalk)
Compressive strength (MPa)	22.5	20.7	12 or more
Flexural strength (MPa)	3.9	3.6	1.2 or more
Compressive strength after 100 cycles of freezing and thawing (%)	87.1	82.4	80 or more
Skid resistance (BPN)	48.3	48.0	30 or more
Permeability coefficient (cm/s)	6.8 × 10^−3^	6.10 × 10^−3^	1.0 × 10^−3^

**Table 9:** NO removal efficiencies according to colloidal silica content.

Sample Code	Efficiency of NO Reduction (%)
T7.5	77.5
T7.5S10	74.4
T7.5 S20	61.8
T7.5 S30	42.8

**Table 10:** NO removal efficiencies according to binder content.

Sample Code	Efficiency of NO Removal (%)
T7.5	77.5
T7.5S10	74.4
T7.5S10B10	72.5
T7.5S10B20	68.4
T7.5S10B30	60.2

**Table 11:** Accelerated weathering performance of TiO_2_-coated permeable concrete.

Item	Value		
	T7.5	T7.5S10	T7.5S10B20
Appearance after accelerated weather resistance evaluation	No creaking, falling and peeling		
Gray scale after accelerated weather resistance evaluation	Grade 4	Grade 4	Grade 4
Color difference after accelerated weather resistance evaluation (ΔE)	2.0	1.5	1.5

**Table 12:** Comparison of NO removal efficiencies before and after accelerated weathering.

Sample Code	Efficiency of NO_x_ Removal (%)		Remarks
	Before AWR	After AWR	
T7.5	77.5	26.0	−51.5%
T7.5S10	74.4	41.9	−32.5%
T7.5S10B20	68.4	54.6	−13.8%

**Table 13:** Nitrate concentrations before and after NO_x_ removal evaluation.

No.	Nitrate Concentration (mg/L)				UV Illuminance (W/m^2^)
	General Pavement		Photocatalyst Coated Pavement		
	Before	After	Before	After	
1st	1.595	1.521	4.712	15.511	11.07
2nd	2.258	2.773	5.596	8.173	2.12

## Data Availability

The original contributions presented in this study are included in the article. Further inquiries can be directed to the corresponding author.

## Appendix A

### Appendix A.1

Figure A1 presents photographs of the accelerated weathering test chamber used in this study. The chamber enables controlled exposure of the coated permeable concrete specimens to UV irradiation, temperature, humidity, and intermittent water spray in accordance with KS M ISO 4892-2:2013. During the 300 h weathering test, the internal conditions were maintained at a black panel temperature of 63 ± 3 °C and relative humidity of approximately 50 ± 10%, with repeated UV irradiation and moisture cycles. This facility allowed simulation of long-term outdoor environmental effects within a shortened timeframe, thereby enabling evaluation of coating durability, discoloration, surface changes, and retention of NOx removal performance.

### Appendix A.2

Figure A2 illustrates the configuration of the outdoor mock-up test-bed used to evaluate the NOx removal performance of the photocatalyst-coated permeable pavement under field-relevant conditions. A sealed test space was constructed using polyethylene (PE) film to enable controlled introduction and retention of exhaust gases while allowing UV transmission for photocatalytic activation. The test-bed consisted of two separate sections: a control area with conventional permeable concrete pavement and a photocatalyst-treated area using TiO_2_-coated eco-friendly permeable concrete (Figure A2b). An exhaust gas generation device fabricated from an aged automobile engine supplied NOx-containing emissions into the chamber (Figure A2c). Real-time NO concentration measurements and system operation were managed from the control room, as shown in Figure A2d. This setup enabled comparative evaluation of photocatalytic NO removal behavior under conditions closely simulating actual roadside environments.
